# A Narrative Review of Maternal and Perinatal Outcomes of Dengue in Pregnancy

**DOI:** 10.7759/cureus.48640

**Published:** 2023-11-11

**Authors:** Shivani Ahuja, Pramita Muntode Gharde

**Affiliations:** 1 Community Medicine, Jawaharlal Nehru Medical College, Datta Meghe Institute of Higher Education and Research, Wardha, IND

**Keywords:** denv, maternal outcomes, perinatal outcomes, pregnancy, dengue

## Abstract

Dengue is one of the most prevalent mosquito-borne diseases in today’s world, especially in India. It is an important health problem and it is very important to address it promptly. Acquiring dengue during pregnancy can have a considerable influence on the health of the mother and baby. In dengue fever, moderate to severe consequences can occur in the mother. Severe dengue poses additional risks to pregnant women due to the likelihood of sequelae such as severe dengue, preeclampsia, gestational hypertension, anemia, maternal death and hemolysis, organ dysfunction, and even death. Concerns about perinatal outcomes in dengue-affected pregnancies have significantly increased. Compared to uninfected mothers, babies born to mothers with dengue are likely to have worse outcomes. Preterm birth and low birth weight are frequently observed in dengue-affected pregnancies, which can have serious effects on the health and development of the child. Complications such as respiratory distress, thrombocytopenia, and jaundice have also been created in the report. Another important consideration is the vertical transmission of dengue virus from mother to fetus. While infection rates can vary, it increases the chances of the virus crossing the placental barrier and harming a developing baby. Early diagnosis, accurate diagnosis, and care are needed to improve maternal and perinatal outcomes in dengue-infected pregnancies. This article discusses early interventions that can help reduce risks.

## Introduction and background

Infectious dengue fever is common in tropical and subtropical regions [1]. The four serotypes of dengue virus (DENV) that make up the Flaviviridae family of viruses are DENV 1, 2, 3, and 4 [2]. Aedes aegypti and Aedes albopictus are the primary carriers of the disease. Dengue fever normally has an incubation period of 3 to 14 days [3]. Clinically, dengue viral infection can manifest itself in a variety of ways, from asymptomatic to severe dengue or dengue shock syndrome which can be lethal [4]. Only 20% of DENV infections cause high temperature and other symptoms like painful joints and muscles, skin rashes, nausea, severe headaches, and others [5]. Determining whether dengue infection during pregnancy is linked to negative fetal outcomes is necessary given that women of reproductive age in dengue-endemic areas are at risk of contracting the disease. Recent reports link maternal DENV infection during pregnancy to preterm birth, low birth weight, stillbirth, and miscarriage [6]. It is important to understand how this disease affects pregnant women. The pest is most attracted to human hosts, and the habit of pregnant women to stay indoors during the day makes them more susceptible to mosquito bites and disease transmission.

## Review

Methodology

**Figure 1:**
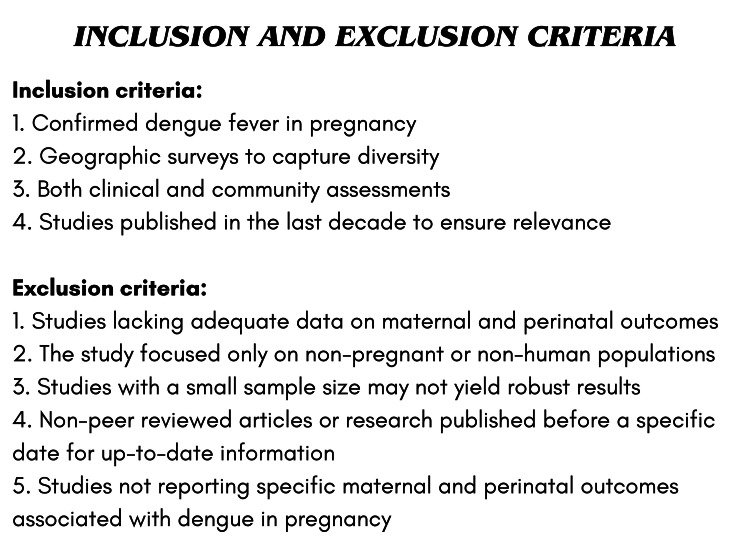
Inclusion and exclusion criteria for assessment of maternal and perinatal outcomes of dengue in pregnancy Credit: Image created by the author

**Figure 2:**
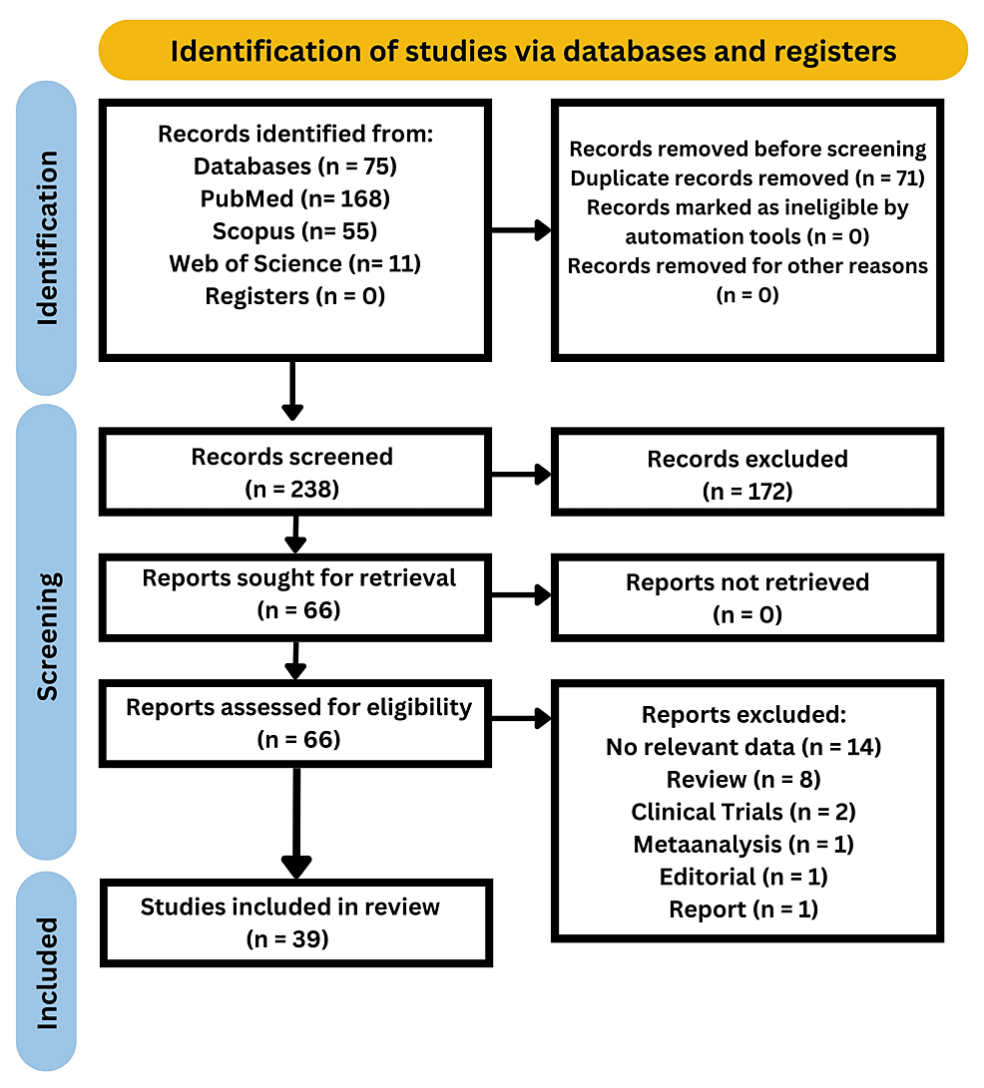
PRISMA methodology used in the study PRISMA: Preferred Reporting Items for Systematic Reviews and Meta-Analyses

A systematic search was done using PubMed, Google Scholar, and various articles. This analysis was performed according to Preferred Reporting Items for Systematic Reviews and Meta-analyses (PRISMA) standards [7]. A thorough literature search was conducted. The phrases (Dengue fever) or (Dengue virus) or (Dengue hemorrhagic fever) or (Dengue shock syndrome) AND (Pregnancy) or (gestational) or (maternal outcome) were associated with the subsequent search terms. There were no language restrictions and studies from all across the world were included. Checking of the reference lists of the included papers and the relevant literature for additional qualifying studies was done. The remaining reports were examined for eligibility using their titles and abstracts after duplicate citations were removed. Based on the inclusion criteria (Figure 1), the titles and abstracts of the papers that made the shortlist were evaluated. Data for each study was extracted and studied which concluded that DENV infection in pregnancy is associated with an increased risk of maternal mortality, stillbirth, and neonatal deaths compared with pregnant women without DENV infection. The method used in the study is depicted below (Figure 2).

Increased temperature with thrombocytopenia during pregnancy causes panic among obstetricians. The main fear is the occurrence of dengue hemorrhagic shock or profuse bleeding. Of the women with dengue fever in pregnancy, primary dengue is seen where both immunoglobulin G and immunoglobulin M are initially negative and paired sera testing taken after 2 weeks is positive [8]. Various effects of dengue might occur during pregnancy. Depending on the severity of the illness, the stage of pregnancy, and the mother's general health, dengue can have mild to severe effects on pregnant women and their unborn children. For determining the risk and treatment of dengue in pregnant women, it is essential to comprehend these elements.

Factors that can influence the severity and outcomes of dengue in pregnancy

An important factor in determining the severity of dengue infection is the immunological response. Serious dengue may be more contagious among pregnant women with compromised immune systems, such as those with pre-existing illnesses or immunosuppressive disorders, such as microchimerism, systemic lupus erythematosus, and rheumatoid arthritis [9]. On the other hand, women who have a strong immune response might have milder symptoms [10]. Results may vary depending on when a pregnant woman contracts dengue. Studies indicate that when compared to infections that happen in the third trimester, dengue infections in the first and second trimesters may carry a greater risk of harmful fetal consequences. On the other hand, severe dengue infection during any stage of pregnancy can have negative effects [11].

The virulence and propensity to produce severe disease can differ among different DENV strains. Some strains may put pregnant women at greater risk for problems and/or more severe symptoms. Pregnant women with underlying health issues, such as diabetes, hypertension, or immunological diseases, may be more likely to have difficulties and develop severe dengue. The body's capacity to combat the illness and deal with its repercussions may be hampered by these disorders [12]. The immunological health of the expectant mother has a big impact on the results of the dengue infection. If infected with the same serotype again, women who have already been exposed to dengue and have built up immunity to it may experience lesser symptoms. However, repeated infections with various serotypes can raise the danger of developing severe dengue [13]. The clinical manifestation and treatment of dengue in pregnancy might be complicated by co-infections with other viruses, such as malaria or the Zika virus. More serious symptoms and negative results could result from these co-infections.

**Figure 3:**
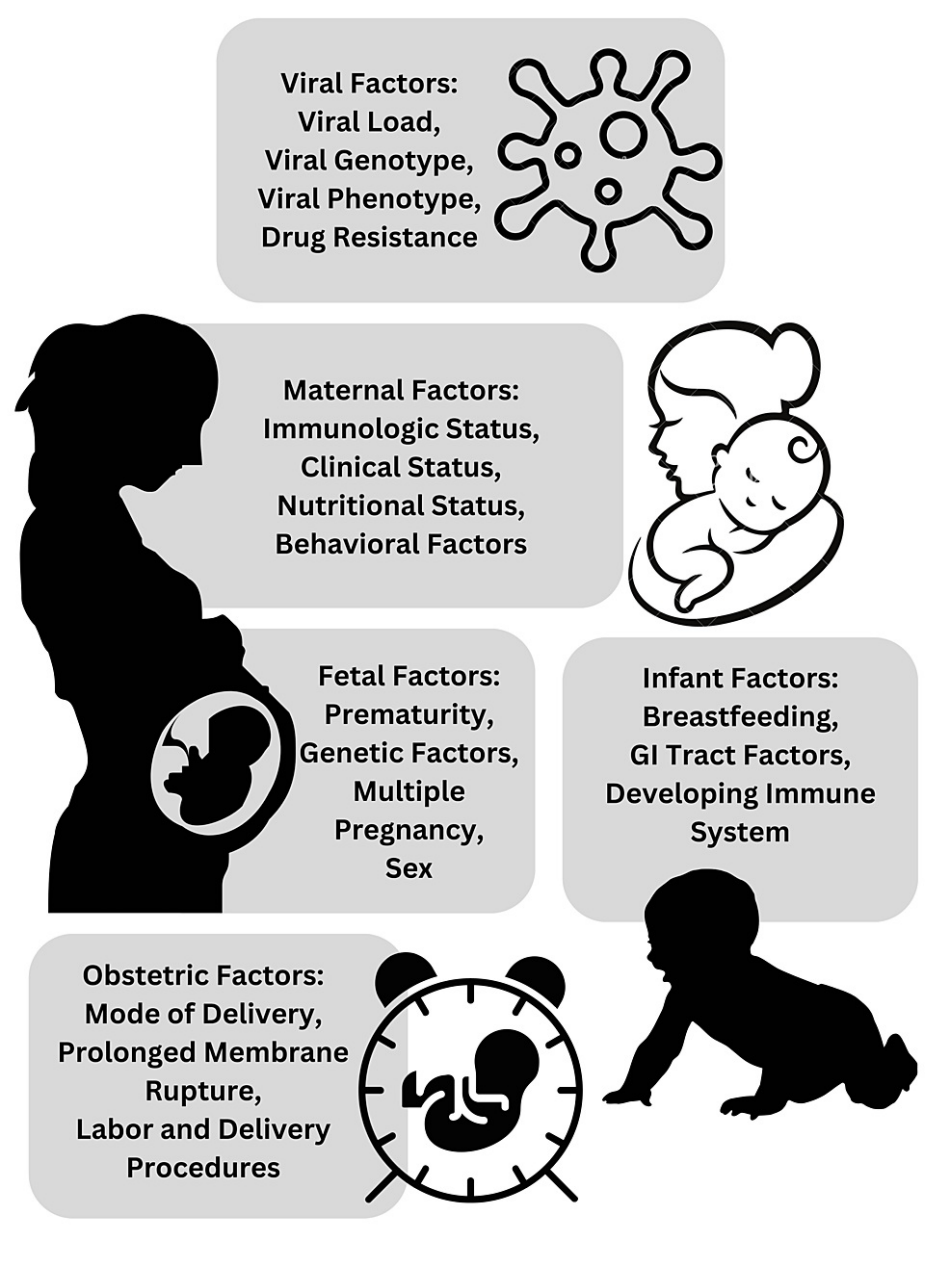
Factors that can influence the severity and outcomes of dengue in pregnancy Credit: Image created by the author GI: Gastrointestinal

Access to timely diagnosis of dengue infection and regular prenatal care can have a big impact on outcomes. Adequate prenatal care enables close health monitoring of the expectant mother and prompt identification of any dengue-related problems. To minimize the risk of problems, dengue in pregnancy must be properly treated and managed medically. The results can be improved with prompt measures such as fluid replacement therapy, pain medication, and careful observation. The community's efficacy in reducing mosquito populations, as well as the area a pregnant woman lives in, can affect the risk of dengue transmission. Thus, viral, maternal, fetal, infant, and obstetric factors influence the severity of dengue infection (Figure 3) [14]. Effective mosquito control, including the removal of breeding sites, the use of insecticides, and personal protective measures, can help reduce the risk of mosquito bites and dengue infection.

Outcomes of dengue in pregnancy

Maternal Outcomes

Nine studies showed that dengue-infected pregnant women have moderate symptoms comparable to those reported in non-pregnant women. Fever, headache, joint and muscle pain, rash, and exhaustion are some symptoms the patient presents with. Most women recover without complications with symptomatic care. In some circumstances, dengue infection can develop into serious conditions like dengue shock syndrome or dengue hemorrhagic fever as can be seen in Table 1 [15]. Dengue can result in irregularities in blood coagulation, which can induce hemorrhage (excessive bleeding). Blood transfusions may be necessary for severe bleeding that affects numerous organs like the digestive tract or the reproductive system. Multiple organs may be affected by severe dengue, which might result in organ dysfunction. The cardiovascular system, liver, and kidneys may all be particularly impacted. Organ malfunction can have negative effects that may call for extensive medical attention.

**Table 1:** Complications of dengue fever affecting pregnant women

Dengue severity	Symptoms and complications	Percentage of women affected	Recovery rate
Dengue fever (DF)	High fever, muscle and joint pain, headaches, rash	17%	High, symptomatic treatment
Dengue hemorrhagic fever (DHF)	High fever, muscle and joint pain, headaches, rash, bleeding, low platelet count, damage to vessels	8%	Varied, can lead to serious complications
Dengue shock syndrome (DSS)	High fever, muscle and joint pain, headaches, rash, shock	7%	Intensive medical care needed

The combination of dengue fever, vomiting, and diarrhea can result in severe fluid loss. Dehydration and electrolyte imbalances, which can harm both the mother and the fetus, are more common in pregnant women. Dengue shock syndrome, which is characterized by a sudden drop in blood pressure, can develop from dengue in specific circumstances. The mother's condition needs to be stabilized right away because shock is a life-threatening condition [16]. Severe dengue infection during pregnancy can cause maternal death, albeit this is rather uncommon. This result is more likely to occur when there is a delay in diagnosis, insufficient medical attention, or consequences including organ failure or significant bleeding. The DENV can cause problems during labor and delivery in 7.2-7.9% of the cases. Dengue-infected pregnant women may have higher bleeding risks and may need close supervision and proper management during labor. A cesarean section delivery may be necessary if there is severe dengue or other problems [17].

Perinatal Outcomes

**Figure 4:**
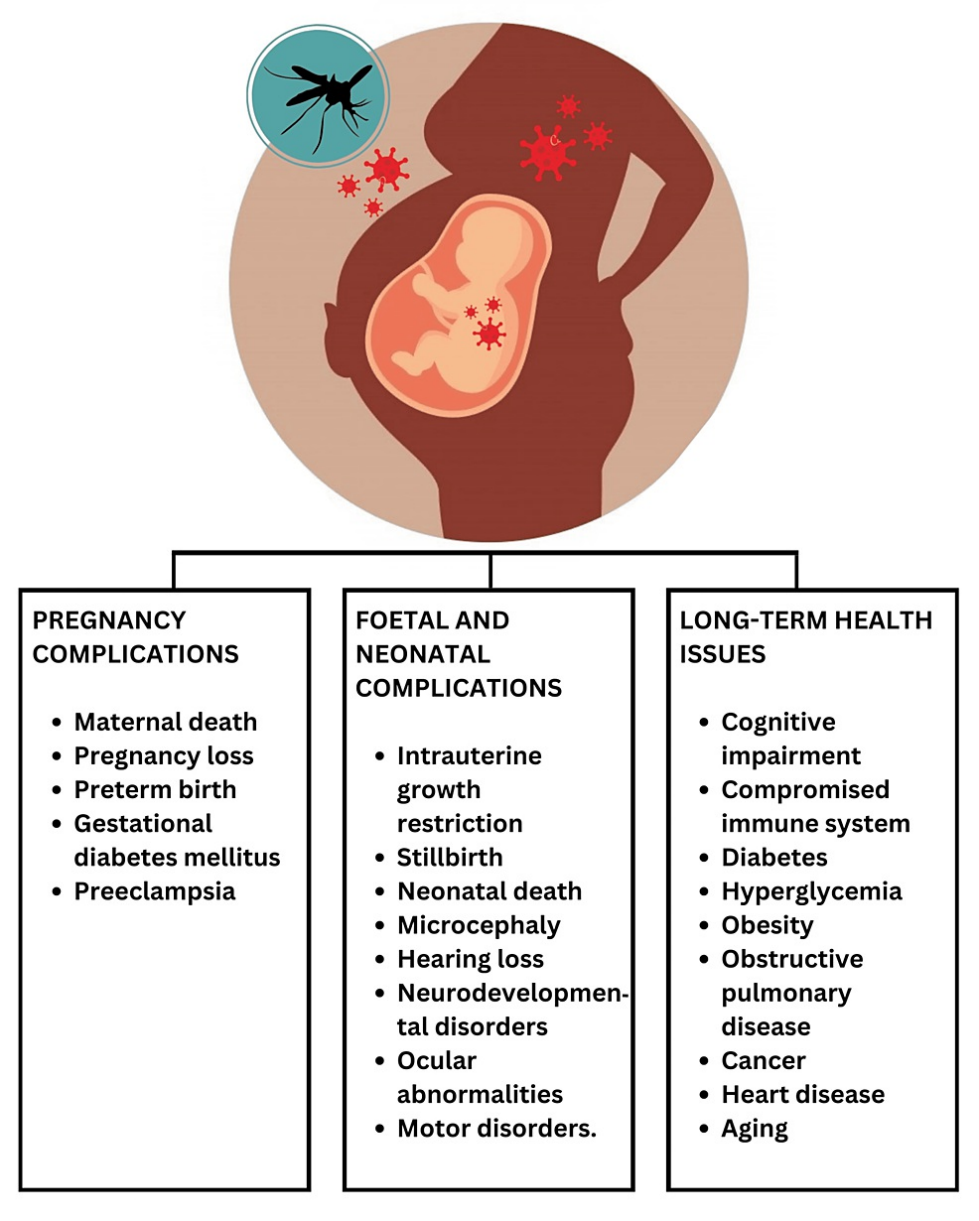
Maternal and fetal complications of dengue Credit: Image created by the author

Eleven studies showed that severe dengue in pregnancy can hamper the normal fetal growth and development of the neonate. This can cause intrauterine growth restriction (IUGR). The children born to IUGR have chronic illnesses, developmental anomalies, and low birth weight. Dengue in pregnancy can cause fetal deaths. The risk of fetal mortality is directly proportional to complications like organ failure and increased bleeding. Dengue can cause premature labor and delivery of the baby [18]. Complications like infections, developmental anomalies, and respiratory distress syndrome are common [19]. Neonatal dengue occurs after the virus crosses the placenta. Neonates and adults present with symptoms like fever, rash, bleeding tendencies, and liver enlargement [20]. Maternal and perinatal factors both lead to complications after DENV infection (Figure 4) [21].

Vertical Transmission

The DENV has the ability to pass through the placenta and infect the circulation of the fetus. This can happen at any point in the pregnancy, but it is more likely to happen when the mother contracts the infection right before giving birth. If the mother is viremic (has the virus in her bloodstream) during labor and delivery, there is a chance that the baby will get the illness from contact with contaminated maternal blood or from passing through the birth canal [22].

Vertical Transmission Influencing Factors

The risk of vertical transmission is greatly influenced by the presence and persistence of maternal viremia (virus in the circulation) [23]. Higher levels of maternal viremia increase the risk of transmission to the fetus. When the mother contracts dengue closer to the time of birth, vertical transmission is more likely to happen. It's crucial to remember that transmission can happen at any point throughout pregnancy. The fetus may receive some protection from maternal antibodies developed as a result of a prior dengue infection or vaccine. These antibodies may lessen the likelihood or impact of vertical transmission [24]. The mother's viral load and strain can affect the likelihood of vertical transmission. The potential for vertical transmission may be higher in some DENV strains. It is thought that 1-6% of dengue cases are transmitted vertically on average [25]. Vertical transmission rates can vary depending on the geographical region, viral strains circulating in the area, and the prevalence of dengue in the population. When a woman has severe dengue or a high virus load, the risk of vertical transmission is typically increased [26].

Repercussions for the Newborn

If a fetus or infant contracts dengue, they may develop neonatal dengue, which manifests as fever, rash, bleeding tendencies, and liver enlargement identical to adult dengue symptoms [27]. Neonatal dengue severity can vary. While some infants with moderate symptoms may recover with supportive care, others with severe dengue may need to be hospitalized and need specialized care. Liver damage, respiratory distress, and blood issues are just a few of the complications that can arise from neonatal dengue [28]. In a study on 120 pregnant women infected with DENV, premature birth and low birth weight were reported in about 10% and 18% of pregnancies, respectively [29].

Management

If left untreated, viral illnesses including dengue, malaria, and many others can even be fatal. Pregnant women should exercise extreme caution and adopt safe practices to safeguard themselves and their unborn children [30]. In pregnancy, it is essential to take extreme precautions and pay close attention to one's nutrition and health [31]. Due to their lowered immunity, expectant mothers are more likely to contract illnesses and infections. Therefore, it is essential to avoid acquiring diseases [32]. Additionally, one should avoid visiting places where there is a significant danger of contracting an infection. If an expecting mother does contract dengue fever, quick care should be given to nutrition and hydration levels. It is important to consume more fluids along with essential salts to keep the body healthy. Important nutrients that you consume during your recovery period balance the embryo's fluid levels, which cater to the baby [33]. The symptoms of dengue during pregnancy are the same as those of a healthy person. However, because of the toll that pregnancy has on your body, the condition's severity can worsen. It is possible to develop a high fever, stomach pain, agonizing headaches, vomiting, and disorientation [34]. The platelet count may drop in case of dengue; therefore, transfusions should be taken into consideration. The condition demands ongoing attention and observation.

Treatment and prevention

Treatment for dengue fever necessitates a lot of liquids, relaxation, and nutrition. Doctors typically recommend paracetamol and anti-inflammatory drugs, which lower body temperature, to treat fever. But if you are expecting, you should always use caution and only take medications if your doctor has given a clean slate [35]. The dosage can occasionally be decreased. It is also possible to try natural remedies to reduce fever and strengthen the immune system [36]. Other effective treatments include using sandalwood paste and sponging with a cool cloth. The death rate can drop to 1% with early discovery and adequate medical care [37]. Since they are most at risk, mothers who contract dengue just days before the due date or shortly after giving birth should be constantly monitored [38]. It's vital to remember, however, that nursing does not prove to be a barrier for new mothers who contract dengue. Breastfeeding does not transmit dengue from the mother to the child. Strong nutrients and antibodies included in a mother's milk can protect a baby against severe diseases, including dengue. However, formula milk may be an alternative to take into account if a mother has a serious infection [39].

## Conclusions

The maternal and perinatal outcomes of dengue infection during pregnancy are a subject of important medical concern. Dengue fever, caused by the mosquito-borne DENV, presents unique challenges for expecting mothers and their unborn children. Dengue in pregnancy can lead to moderate symptoms such as fever, headache, muscle pain, and rash. Severe dengue can cause irregularities in blood coagulation, leading to hemorrhage and organ dysfunction. Dengue fever, vomiting, and diarrhea can result in severe fluid loss, dehydration, and electrolyte imbalances, which can be harmful both to the mother and the fetus. Severe dengue infection during pregnancy can cause maternal death, but this is rare. Perinatal outcomes include obstetric complications, such as IUGR, chronic illnesses, developmental anomalies, and low birth weight. Neonatal dengue occurs after the virus crosses the placenta, presenting symptoms like fever, rash, bleeding tendencies, and liver enlargement.

To manage dengue fever, pregnant women should exercise extreme caution and adopt safe practices such as preventing mosquito bites and eliminating breeding sites. They should avoid acquiring diseases and visiting places with high infection risks. Treatment for dengue fever requires fluids, relaxation, and nutrition. Doctors typically recommend paracetamol and anti-inflammatory drugs, but pregnant women should exercise caution and take medications if prescribed by their doctor. In our study, we focused on downstream immediate maternal and perinatal outcomes of dengue in pregnancy, shedding light on its impact on pregnancy. However, continued research is needed to examine several important aspects to better understand this complex issue.
